# The Administration of Cannabinoid Receptor 2 Agonist Decreases Binge-like Intake of Palatable Food in Mice

**DOI:** 10.3390/ijms26051981

**Published:** 2025-02-25

**Authors:** Luis Miguel Rodríguez-Serrano, María Elena Chávez-Hernández

**Affiliations:** Facultad de Psicología, Universidad Anáhuac México, Universidad Anáhuac Avenue #46, Lomas Anáhuac, Huixquilucan 52786, Mexico; elena.chavez@anahuac.mx

**Keywords:** CB2 cannabinoid receptor, binge-like intake, palatable food, adolescent mice

## Abstract

Binge eating disorder (BED) is characterized by uncontrollable episodes of eating in a short period of time, with a subjective loss of control of overconsumption behavior. The role CB2 cannabinoid receptor (CB2R) plays in binge-like intake has not yet been identified. In this regard, the present study aims to evaluate the effect of the administration of CB2R agonist, antagonist, or both on binge-like intake of palatable food (PF) in adolescent mice. We used 35 C57BL6/J male mice of 30 postnatal days in this research; all animals were housed individually and had ad libitum access to a standard diet (SD) and water. Animals were evaluated for a total of 15 sessions of the Binge Eating Test (BET), which consisted of 1 h access to PF (chocolate sandwich cookies) according to intermittent diet protocol, with one-day access/one-day no-access. PF and SD caloric intake, as well as the PF binge index (defined as consuming ≥20% of total caloric intake per day during the 1 h access to PF), were analyzed. Mice were randomly assigned to one of the following treatment groups: (1) control; (2) vehicle; (3) HU308, selective CB2R agonist; (4) AM630, selective CB2R antagonist; (5) AM630+HU308 coadministration of antagonist and agonists of CB2R. All treatments were administered intraperitoneally before BET sessions. Our results show that HU308 significantly reduced binge-like intake of PF, while no significant differences were found in the rest of the groups. These results suggest that activation of the CB2R decreases the binge-like intake in adolescent mice and that chronic overconsumption in conditions of non-homeostatic feeding can be modulated by the CB2R. Furthermore, the activation of CB2R may also modulate reward pathways, reducing binge-like behavior, which could be further explored in future studies as a treatment for BED.

## 1. Introduction

Binge eating disorder (BED) is characterized by uncontrollable episodes of eating in a short period of time, with a subjective loss of control and overconsumption behavior [1,2]; it is also the most prevalent of eating disorders [3,4]. Additionally, in BED, overconsumption of palatable food (PF) suggests that the behavior is driven by the rewarding properties of food rather than homeostatic signals [5]. Furthermore, adolescents with BED are at increased risk of being overweight and obese [6]. Food intake involves metabolic and nutritional signals that regulate homeostatic eating and reward signals that code pleasurable aspects of food that drive hedonic consumption [7]. In this regard, an equilibrium is important between eating to provide energy consumption for homeostatic functions and reward-induced overconsumption [8].

The endocannabinoid system (ECS) plays a major role in the regulation of food intake, both centrally and in the periphery [1]. The ECS comprises the endogenous ligands anandamide (AEA) and 2-arachydonyl glycerol (2AG) [9,10], as well as the enzymatic machinery in charge of synthesis and degradation. AEA is synthesized from phosphatidylethanolamine and degraded by fatty-amino-acid-hydrolase (FAAH) [11], while 2AG is synthesized from phosphatidylinositol and degraded by the enzyme monoacylglycerol-lipase (MAGL) into arachidonic acid and glycerol [12]. Both AEA and 2AG are synthetized by postsynaptic neurons in response to neurotransmitter release, functioning as negative feedback regulators that inhibit further neurotransmitter release. Thus, ECS is a retrograde messenger system that inhibits neurotransmitter release at excitatory or inhibitory synapses [8,13]. Additionally, the ECS includes two cannabinoid receptors: Cannabinoid Receptor 1 (CB1R) and Cannabinoid Receptor 2 (CB2R). In particular, the CB2R is expressed on dopaminergic cell bodies at the ventral tegmental area (VTA) [14,15] and nucleus accumbens (NAc) [7,16]. Furthermore, it has been suggested that CB2R is mainly expressed in the postsynaptic [17]. Regarding this, activation of CB2R reduces intracellular cAMP levels and enhances M-type K^+^ channel function, leading to a reduction in the excitability of VTA neurons [18]. 

When CB2R is activated, it couples to Gi/o proteins, with more affinity to Gi than to Go, and their activation is associated with different cellular pathways, including adenylate cyclase (AC), cAMP, protein kinase A (PKA), ERK 1/2, p38 mitogen-activated protein kinase (MAPK p38) and AKT [19,20]. Specifically, the activation of CB2R results in the inhibition of AC [19,20,21], the cAMP/PKA dependent pathway [20], and activation of intracellular kinases, such as PI3K-Akt pathway, and extracellular signal-regulated (ERK) kinases, which ultimately results in the suppression of neuronal activity [21]. Furthermore, CB2R may modulate mesolimbic dopaminergic neurons and participate in cocaine self-administration behavior [15] and sugar consumption [7].

Research shows there is a clear relationship between ECS and BED. It has been identified that binge-like intake in stress and repeated food restriction induces the down-regulation of FAAH [22] and up-regulated gene expression of CB1R in the hypothalamus in female rat adults [23]. Furthermore, CB2R has been found in brain areas that are part of the reward system and are involved in food intake regulation [1,24]. Also, CB2R has been identified in dopamine neurons, which may play important roles in the modulations of psychomotor behaviors, anxiety, depression, and the rewarding effects of alcohol, cocaine [25] and methamphetamine [26]. Furthermore, it has been shown that reduced CB2R signaling by administration of antagonists leads to increased intake of standard diet in food-deprived mice [27]. In this regard, Amancio-Belmont et al. [28] show that there is an age-related expression of CB2R in the prefrontal cortex (PFC), NAc, and Hippocampus (Hipp), where adolescent rats show lower expression of CB2R than adult and aged rats which may be related to motivation and decision-making modulation.

Studies indicate there is a clear role of CB2R in food intake regulation. For example, increased CB2R expression in the hypothalamus and high-fat food intake has been reported to be induced by maternal high-fat diet in male and female rat offspring at weaning and adulthood [29]. Furthermore, overexpression of CB2R induces food addiction after exposure to PF in mice [30]. Also, Bourdy et al. [7] show that high sugar intake is induced by an increased expression of CB2R in the NAc, while Bi et al. [31] report that administration of a selective CB2R agonist produced a reduction in sucrose self-administration in mice adults. Additionally, Amancio-Belmont et al. [28] also report that adolescent rats, who express less CB2R in the PFC, Nacc, and Hipp, ingest significantly more chocolate pellets in a fixed ratio and progressive ratio paradigm than adult and aged rats, showing a higher motivation to obtain a reinforcer. 

Recently, the role of CB2R in binge intake has been identified [1]. However, few studies have evaluated the role CB2R has in the modulation of binge-like intake of PF in adolescent mice, which is important given that CB2R expression undergoes changes in an age-related manner and may be involved in motivation for PF intake. Here, we evaluate the effect of the administration of CB2 cannabinoid receptor agonist, antagonist, or both on binge-like intake of PF in adolescence in mice. 

## 2. Results

### 2.1. Increased Palatable Food Intake from Binge Eating Tests One Through Nine

**Figure 1:**
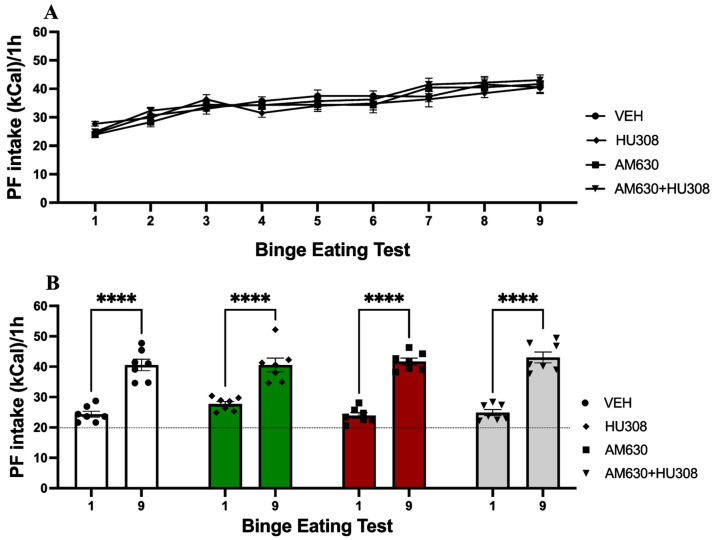
Palatable food (PF) kilocaloric (kCal) intake in the Binge Eating Test. (**A**) Point-plot showing the increase of PF intake in Binge Eating Test 1 through Binge Eating Test 9. (**B**) Bar graphs showing the comparative PF intake in Binge Eating Test 1 versus Binge Eating Test 9. Data express the mean ± SEM (n = 7/group). **** *p* < 0.0001. Two-way ANOVA analysis with Šidák’s multiple comparison test.

The behavioral binge-like intake of PF was evaluated in adolescent mice starting Binge Eating Test (BET) sessions on postnatal day (PND) 30. BET sessions evaluate overconsumption of PF in a short time with the intermittent access model [32,33], with access to PF on the following days: Monday, Wednesday, and Friday for one hour (11–12 h). As shown in Figure 1A, PF intake increased in BET across experimental weeks. Two-way ANOVA analysis shows a significant effect of time (F_(3.54, 21.26)_= 43.67; *p* < 0.0001), with 52.63% of variance explained, and no significant effect of group (F_(1.187, 13.12)_ = 0.6264; *p* = 0.5639), or interaction (F_(3.821,22.92)_ = 0.8965; *p* = 0.4784) in PF intake in BET one through nine. Additionally, to determine changes in consumption in the BET sessions, we made a comparative analysis of binge intake in BET sessions one versus nine. In this regard, Two-way ANOVA analysis with repeated measures was conducted to evaluate differences of PF intake between BET one and nine revealing significant differences between both (F_(1, 6)_ = 616.2, *p* < 0.0001), with 82.38% of variance explained, and no significant effect of group (F_(3,18)_ = 0.4189; *p* = 0.7416) or interaction (F_(3,18)_ = 2.108; *p* = 0.1349). Post hoc analysis with the Šidák test showed significant differences (*p <* 0.0001) between BET one and nine in all experimental groups, as shown in Figure 1B. These results suggest that there is an increasing preference for PF intake during each BET, evidencing a heightened binge-like behavior in mice.

### 2.2. The Administration of CB2R Agonist Modifies Binge-like Intake of Palatable Food

**Figure 2:**
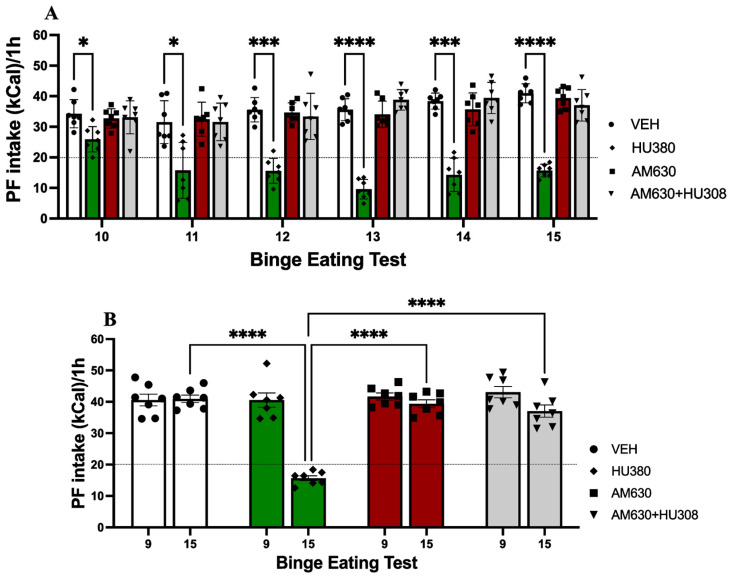
Palatable food (PF) kilocaloric (kCal) intake in the Binge Eating Test under the effect of CB2R agonist and/or antagonist. (**A**) Bar graph shows a bar plot of PF intake of Binge Eating Test 10 through Binge Eating Test 15 (under the effect of agonist and/or antagonist). (**B**) Bar graph shows a bar plot of PF intake of Binge Eating Test 9 (without treatment) versus Binge Eating Test 15 (last under the effect of agonist and/or antagonist). Data express the mean ± SEM (n = 7/group). * *p* < 0.05, *** *p* < 0.001, **** *p* < 0.0001.

We evaluated the intraperitoneal administration of an agonist and antagonist of CBR2 or both in the binge-like intake of PF. As shown in Figure 2A, intraperitoneal injection of HU308 significantly reduces binge-like intake of PF from administrations ten to fifteen. Two-way ANOVA with repeated measures on the BET revealed a significant effect of time (F_(2.868, 17.21)_ = 4.069; *p* = 0.0248) explaining 3.105% of variance, group (F_(1.192, 8.95)_ = 100.1; *p* < 0.0001) with 68.55% of variance explained, and interaction (F_(3.819, 22.91)_ = 4.77; *p* = 0.0065) explaining 8.359% of variance. Post hoc Šidák’s analysis showed significant differences between HU308 and vehicle groups in BET 10 to 15 (all *p* values < 0.05). Furthermore, to evaluate changes in PF binge-like intake before treatment and the last BET under treatment, we compared BET 9 (before CB2R agonist/antagonist administration) and 15; the results are shown in Figure 2B. Two-way ANOVA analysis indicates a significant difference between both BET sessions (F_(1,6)_ = 78.91; *p* = 0.0001) explaining 19.75% of variance, as well as a significant effect of group (F_(3,18)_ = 31.41; *p* < 0.0001) with 33.54% of variance explained, and interaction (F_(3,18)_ = 21.01); *p* < 0.0001) explaining 28.54% of variance. Post hoc Šidák’s analysis indicates a significant difference in BET 15 between HU308 and VEH, AM630, and HU308+AM630 groups (all *p* values < 0.0001). These results indicate that the administration of a CB2R agonist reduces binge-like intake of PF.

### 2.3. Body Weight Increases Across Binge Eating Tests

**Figure 3:**
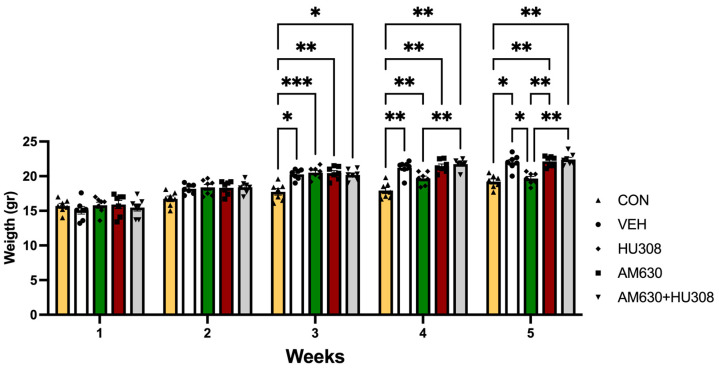
Body weight. Bar graph shows the bar plot of body weight per week. Data express the mean ± SEM (n = 7/group). * *p* < 0.05, ** *p* < 0.01, *** *p* < 0.001.

Two-way ANOVA analysis was used to evaluate the differences between groups on mean weekly body weight. The results of the analysis indicate a significant effect of time (F_(2.697, 16.18)_ = 299.4; *p* < 0.0001) explaining 66.31% of variance, group (F_(2.419, 14.51)_ = 15.60; *p* = 0.0001) with 11.32% of variance explained, and interaction (F_(4.074, 24.44)_ = 5.454; *p* = 0.0027) explaining 7.217% of variance (see Figure 3). 

Šidák’s multiple comparisons test revealed significant differences in week 3 between CON and VEH groups (*p* = 0.0443), CON and HU308 groups (*p* = 0.0011) and between CON and AM630 (*p* = 0.0051), in week 4 between CON and VEH groups (*p* = 0.0142), CON and HU308 (*p* = 0.003), CON and AM630 (*p* = 0.017), CON and AM630+HU308 groups (*p* = 0.0023), and in week 5 between CON and VEH groups (*p* = 0.0341), CON and AM630 (*p* = 0.01), CON and AM630+HU308 groups (*p* = 0.0064), HU308 and AM630 (*p* = 0.0073), and HU308 and AM630+HU308 groups (*p* = 0.0126).

It is important to note that, even though changes in body weight could also be related to normal animal growth (given that the experiment started in adolescence [PND 30] and continued throughout adulthood [PND 62]), differences found in body weight on weeks 4 and 5 suggest that CB2R agonist group showed reduced body weight similar to the CON group. This finding suggests that treatment with CB2R agonists may have an impact on body weight regulation, which could be modulated by regulating feeding behavior or metabolic processes.

## 3. Discussion

The present study aimed to evaluate the effect of the administration of CB2R agonist, antagonist, or both on binge-like intake of PF in adolescence in mice. Results show that the administration of CB2R agonist significantly reduces binge-like intake of PF compared to control, CB2R antagonist, and coadministration of CB2R agonist and antagonist. This result suggests that chronic systemic activation of CB2R reduces binge-like intake of PF. CB2R has been identified in brain areas that are part of the reward system, such as NAC [7,16], and in areas that are involved in food intake regulation [1,24]. Furthermore, studies suggest that CB2R activity modulates mesolimbic dopaminergic neurons [7]. This modulation may participate in cocaine self-administration behavior and sugar consumption [7,15]. Additionally, we show that the administration of a selective CB2R agonist produced a reduction in sucrose self-administration in mice adults [31]. This may be regulated by reduced excitability of VTA neurons, decreased intracellular cAMP levels, and enhancement of M-type K^+^ channel function that results from CB2R activation [18]. Our results show no significant reduction of binge-like intake by the administration of CB2R antagonist, suggesting that the activity of CB2R is key in reducing this behavior.

It has been reported that CB2R knock-out mice show a significant reduction of chocolate pellets self-administration reinforcements in an operant condition paradigm, while mice overexpressing CB2R reduced responding in the early period but increased in the late period, thus suggesting that the lack of CB2R may constitute a protective factor while overexpression may be a vulnerability for the development of food addiction [30]. Our results show the effects of CB2R activation on binge-like intake of PF in a non-operant paradigm. Furthermore, it is important to note that Garcia-Blanco et al. [30] report significant differences from FR 5:1 operant session 54, while in our study, six BET sessions in the animals’ home cage were sufficient to show a significant reduction in binge-like intake by CB2R agonism, starting on the first BET session. Furthermore, an operant condition paradigm implies that animals are food deprived, while in our study, animals have ad libitum access to SD. In this context, our findings support the use of this model to reliably induce binge-like intake of PF in a non-caloric restriction-dependent manner, while usually caloric restriction is used to increase motivation for binge-like intake [34,35]. Herein, it has been shown that this model of binge-like intake produces powerful changes in overconsumption of PF. Over a nine-session BET, we observed a significant increase in binge-like intake episodes, with PF consumption escalating from 20% to 40% of total daily caloric intake. These results highlight the progressive nature of the behavior when chronically exposed to PF and evidence the potential utility of this model in studying BED under homeostatic conditions without the need for caloric restriction, showing the driving of this maladaptive eating behavior. Furthermore, we show that the development of binge intake of PF in adolescent mice is not dependent on caloric restriction, which is, per se, a risk factor in the development of obesity in adults [36]. Additionally, PF may induce changes in neuronal plastic in the brain reward circuitry that lead to overconsumption [37]. 

The role of CB2R stimulation by endogenous and exogenous ligands leads to an anti-inflammatory response that has consistently been reported [38,39,40]. For example, CB2R activation in microglial cells is associated with increased expression of anti-inflammatory factors such as IL-10 [41]. Furthermore, Wu et al. [42] show that it reduces weight gain, relieves glucose tolerance, enhances insulin sensitivity, and attenuates inflammation by suppressing M1 macrophage polarization in a mice model of obesity. In relation to this, our results show that administration of a CB2R agonist reduces weight gain, which is consistent with prior studies [42,43]. 

In summary, our findings suggest that CB2R participates in the regulation of the consumption of PF and in the mediation of energy balance. This suggests that the activation of CB2R further modulates the reward pathways, reducing binge-like behavior, which could help attenuate patterns characteristic of BED, such as excessive reward-seeking and compulsive consumption. The results from our preclinical study indicate that CB2R modulation presents as a potential novel treatment for BED, which could help attenuate the heightened reward sensitivity that is often observed in individuals with BED. In this regard, CB2R-targeted therapies for BED could address behavioral and neural mechanisms of the disorder, which could be further explored in studies with human subjects. 

## 4. Materials and Methods

### 4.1. Subjects

Thirty-five male C57BL/6J mice adolescents of 30PND. All animals were individually housed to have a precise measure of food intake per animal during the experiment in a temperature (20 °C) and humidity-controlled vivarium on a standard 12:12 light–dark cycle and had ad libitum access to a standard diet (SD; Nutricubos Purina^®^; Vevey, Switzerland; 3.36 kcal/g; 23.0% protein, 3.0% fat and 6.0% fiber) and water. 

### 4.2. Ethical Considerations

All animals used and all experimental procedures in this study were handled in accordance with the guidelines of the Mexican Official Norm NOM-062-ZOO-1999, as well as the international guidelines Guide for the Care and Use of Laboratory Animals of the National Institutes of Health. Furthermore, the project was approved by the Dirección de Investigación, Universidad Anáhuac México with the ID number PI0000154. 

### 4.3. Evaluated Behavior: Binge Eating Test

At PND 25, animals were individually housed and left undisturbed for habituation until starting the experimental protocol at PND 30. All animals had ad libitum access to SD and were manually recorded every 24 h. 

The Binge Eating Test (BET) is defined as a short period of time (1 hour) where animals are exposed to PF (chocolate sandwich cookies, Oreo^®^ Cookies Nabisco^®^ 4.67 kcal/g; 4.1% protein; 19.2% fat; 69.5% carbohydrates) in their home cage under conditions that are not necessarily driven by caloric need, given that animals are not food deprived. In BET sessions, we evaluated the binge-like intake of PF. The BET was evaluated with an intermittent model [32,33], with the following access to PF days access: Monday, Wednesday, and Friday for one hour (11–12 h). Previous studies [32] show that nine sessions are enough to induce and establish binge-like intake of PF. In this regard, we sought to identify the increase in binge-like intake in nine sessions. Furthermore, we evaluated six additional sessions under the effect of treatments to determine changes in behavioral binge-like intake. 

All animals were exposed to a total of 15 BET distributed in five experimental weeks as follows: sessions 1 to 9 (weeks 1 to 3) were baseline BET sessions without treatment, and sessions 10 to 15 (weeks 4 and 5) were BET under treatment according to the experimental group assigned. At the end of each week, animals were weighed to identify the changes in body weight from BET. 

The PF was weighed before and after the 1 h access to register consumption and caloric intake was calculated as follows for both PF and SD: 

*Caloric intake = (WF_found_ − WF_placed_)* × *Kcal*, where *WF_found_* represents the weight in grams of the food found on the cage, *WF_placed_* is the weight of the food when first placed on the cage, and *Kcal* is the kilocalories per gram of PF or SD [32]. Additionally, binge-like intake was determined as consuming ≥20% of total daily kilocalories from PF [44]. First, total caloric intake (TOTAL_kcal_) was calculated as the sum of kilocalories from PF and kilocalories from SD (TOTAL_kcal_ = PF_kcal_ + SD_kcal_); afterward, the proportion of PF intake kilocalories (Kcal %) was calculated as follows [32]:

$$PF\;intake\;kcal\;\%=\left(\frac{PFkcal}{TOTALkcal}\right)\times 100$$

### 4.4. Experimental Design

**Figure 4:**
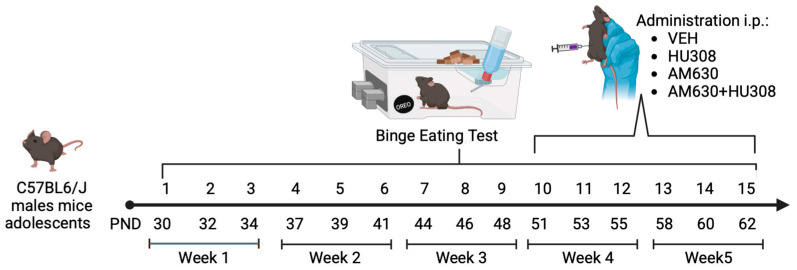
Experimental overview. Experimental timeline: Starting on PND 30, mice were evaluated in 15 Binge Eating Tests distributed across five experimental weeks. VEH, HU308, AM630, and AM630+HU308 groups had access to PF for 1 h for three days per week in the intermittent access protocol. Figure created with BioRender.com ^®^.

Figure 4 shows the experimental overview. To evaluate the effects of administration intraperitoneal of agonist, antagonist, or both CBR2 in binge-like intake of PF, animals were distributed in the following treatment groups (n = 7 for each group):

- CON group, control intact group with no access to PF and BET;
- VEH group, administered vehicle consisting of DMSO and saline solution (1:9; 1 mL/kg) immediately before BET sessions;
- HU308 group, administered selective CB2R agonist HU308 (5mg/kg; Sigma-Aldrich, St. Lous, MO, USA; [45], immediately before BET sessions;
- AM630 group, administration of a selective antagonist of CB2R AM630 (5mg/kg; Sigma-Aldrich, St. Lous, MO, USA; Verty et al. [43]) 15 min before BET sessions;
- AM630+HU308 group, with coadministration of AM630 and HU308, with AM630 injected 15 min before BET, followed by HU308 immediately before BET sessions.

### 4.5. Statistical Analysis

Data were prepared in Excel and are reported as the mean ± standard error of the mean (SEM). Results were analyzed using GraphPad Prism^®^ version 9.3.1 (350) (Graphpad Software LLC, Boston, MA, USA, 2021). Figures were made in GraphPad Prism^®^.

PF binge-like intake is represented as the mean proportion of PF kilocaloric (kcal) intake per BET session, and body weight is represented as the mean weight per week in grams. A two-way ANOVA (group × BET sessions) with Šidák’s multiple comparisons test (∝ < 0.05) was conducted to compare the main effects of treatment group (group) and time (BET sessions), as well as their interaction effects on body weight and binge-like intake during the experiment from BET sessions 1 to 9, to compare between BET sessions 1 vs. 9, BET sessions 10 to 15 (under treatment) and between BET sessions 9 vs. 15.

## Data Availability

The raw data supporting the conclusions of this article will be made available by the authors on request.
